# The Electronic Mental Wellness Tool as a Self-Administered Brief Screening Instrument for Mental Disorders in the General Spanish Population during the Post-COVID-19 Era

**DOI:** 10.3390/ijerph20043204

**Published:** 2023-02-11

**Authors:** Ismael Martinez-Nicolas, Cale Basaraba, David Delgado-Gomez, Olatz Lopez-Fernandez, Enrique Baca-Garcia, Milton L. Wainberg

**Affiliations:** 1 Faculty of Life Sciences, Catholic University of Murcia (UCAM), 30107 Guadalupe, Spain; 2 Sistema Español de Notificación en Seguridad en Anestesia y Reanimación (SENSAR), IDEhA Simulation Centre, Fundación Alcorcon University Hospital, 28922 Alcorcon, Spain; 3 New York State Psychiatric Institute, Columbia University, New York, NY 10024, USA; 4 Department of Statistics, University Carlos III of Madrid, 28903 Getafe, Spain; 5 Santander Big Data Institute, Universidad Carlos III de Madrid, 28903 Getafe, Spain; 6 Department of Psychiatry, University Hospital Jimenez Diaz Foundation, 28040 Madrid, Spain; 7 Faculty of Psychology, Madrid Complutense University, 28049 Madrid, Spain; 8 Faculty of Psychology, Francisco de Vitoria University, 28049 Madrid, Spain; 9 Faculty of Psychology, Cardenal Cisneros Centro de Estudios Universitarios, 28223 Madrid, Spain; 10 Department of Psychiatry, University Hospital Rey Juan Carlos, 28933 Móstoles, Spain; 11 Department of Psychiatry, General University Hospital of Villalba, 28400 Collado Villalba, Spain; 12 Department of Psychiatry, University Hospital Infanta Elena, 28342 Valdemoro, Spain; 13 Department of Psychiatry, Madrid Autonomous University, 28049 Madrid, Spain; 14 CIBERSAM (Centro de Investigacion en Salud Mental), Carlos III Institute of Health, 28029 Madrid, Spain; 15 Faculty of Psychology, Universidad Catolica del Maule, Talca 3605, Chile; 16 Department of Psychiatry, Centre Hospitalier Universitaire de Nîmes, 30900 Nîmes, France

**Keywords:** diagnostic screening programs, mental health, primary health care, secondary health care, reproducibility of results, mental disorders, eHealth, mHealth, mobile app

## Abstract

(1) Background: In the “post-COVID-19 era”, there is a need to focus on properly assessing and addressing the extent of its well-established mental health collateral damage. The “Electronic Mental Wellness Tool” (E-mwTool) is a 13-item validated stepped-care or stratified management instrument that aims at the high-sensitivity captures of individuals with mental health disorders to determine the need for mental health care. This study validated the E-mwTool in a Spanish-speaking population. (2) Methods: It is a cross-sectional validation study using the Mini International Neuropsychiatric Interview as a criterion standard in a sample of 433 participants. (3) Results: About 72% of the sample had a psychiatric disorder, and 67% had a common mental disorder. Severe mental disorders, alcohol use disorders, substance use disorders, and suicide risk had a much lower prevalence rate (6.7%, 6.2%, 3.2%, and 6.2%, respectively). The first three items performed excellently in identifying any mental health disorder with 0.97 sensitivity. Ten additional items classified participants with common mental disorders, severe mental disorders, substance use disorders, and suicide risk. (4) Conclusions: The E-mwTool had high sensitivity in identifying common mental disorders, alcohol and substance use disorders, and suicidal risk. However, the tool’s sensitivity in detecting low-prevalence disorders in the sample was low. This Spanish version may be useful to detect patients at risk of mental health burden at the front line of primary and secondary care in facilitating help-seeking and referral by their physicians.

## 1. Introduction

Mental and psychological diseases have been identified as the world-leading causes of years lived with disability [1]. Indeed, people with mental health problems around the world have limited access to psychological care [2,3,4]. Developing countries face vast challenges in terms of financing these types of diseases and mental health service availability [5,6]. In low- and middle-income countries, more than 75% of people identified with serious anxiety, problematic mood changes, impulse control, or substance abuse disorders did not receive any traditional or digital care [7,8,9]. Even developed countries such as the United States (U.S.) also face mental health access problems with nearly 40% of the population residing in areas where no mental health professionals are available [10]. For these reasons, it is urgent to start implementing screening procedures to detect possible mental health burdens in patients who attend health centers that facilitate referral to psychological or psychiatric services.

Internet medical care and telehealth have profoundly changed access and the mode of seeking physical and mental health care, yet only in settings where the proper infrastructure exists [11]. Similarly, digital technology is increasingly being used to address the mental health access crisis worldwide [12,13], albeit mostly focused on addressing depression and anxiety symptoms [9,14,15]. Telehealth should overlap with public health strategies to greatly improve the large mental health treatment gap to increase access and enhance the ability of community medical staff to identify and manage psychological problems, especially after the COVID-19 pandemic [2,16,17,18,19,20].

Their underrated importance in public health is now highlighted by the impact of the COVID-19 pandemic [21]. During the pandemic, many factors have been identified as contributors to widespread emotional distress in the general population. It has led to both a higher prevalence of mental health and substance use symptoms and disorders globally, and recurrences or worsening of symptoms among people with mental disorders [22]. Studies involving patients have shown higher levels of depression, anxiety, and sleep disturbances in these individuals during strict lockdown measures in many countries that have flexibly managed hospital practices during pandemic times [23,24,25,26], including Spain [27,28]. Therefore, in this “post-COVID-19 era”, it is necessary to properly assess and address the extent of this mental health collateral damage and the usage of mobile health (mHealth) tools to cover the reported mental health services gap.

At this preliminary stage of emerging mHealth tools after the outbreak of the COVID-19 pandemic, there is a need for specific mHealth screening tools to sensibly detect those patients who attended healthcare settings with potential mental health problems. These mental health problems can be unnoticed by physicians’ consultations or other health professionals who are not psychiatrists or clinical psychologists.

One such advance which covers a gap in current screening instruments is the “Electronic Mental Wellness Tool” (E-mwTool) [29], which was developed and validated in a low-income country, Mozambique (Africa [2,30]). Thus, it has not been tested in developed countries or languages different from Portuguese. It is a brief screening and triage tool that could provide a substantial benefit to healthcare systems in low-, middle-, and high-income countries.

The original E-mwTool is a 12-item instrument designed as a stepped- or stratified-care management approach to improve access to care with rigor. The E-mwTool supports nonpsychiatric specialists in primary and secondary care settings, such as general practitioners (GPs) and specialist physicians, among other allied health professionals in community settings, in screening patients for mental disorders [29]. In the original setting, the first three items identified any mental disorder with excellent sensitivity (94%), and nine additional items further classified individuals into four treatment categories (i.e., common, severe, substance use disorders, and suicide risk) with high specificity (63%–93%) [29].

However, the original E-mwTool does not include items screening for substance use disorder because of low prevalence in the sample used for development. Accordingly, we will include one more single-item measure in our study regarding illicit drug use and nonmedical use of prescription drugs [31] to facilitate addiction detection in healthcare settings. As a result, we will examine the performance of an updated E-mwTool comprising 13 items.

Along with this screening tool, five internationally validated, self-administered screening tests were employed: the Alcohol Use Disorders Identification Test (AUDIT-3 [32]), Columbia-Suicide Severity Rating Scale (C-SSRS [33,34]), Generalized Anxiety Disorder-7 (GAD-7 [35]), and Patient Health Questionnaire-9 (PHQ-9 [36]). Altogether, the Spanish E-mwTool with 13 items or these self-administered questionnaires could provide identification and further orientation for patient referral from physicians to specialized mental healthcare units.

Therefore, the primary purpose of the current study was to examine the validity of the 13-item E-mwTool against the Mini International Neuropsychiatric Interview (MINI-Plus) diagnoses as the criterion standard in a Spanish population referred for psychiatric consultation. A secondary aim was to examine the performance of the aforementioned five self-administered questionnaires in a Spanish population to inform further use of these screening tools in mHealth settings.

## 2. Materials and Methods

### 2.1. Study Design

We conducted a cross-sectional validation study on mental health patients from Madrid (Spain). This paper is compliant with the Standards for the Reporting of Diagnostic Accuracy Studies (STARD) statement [37].

### 2.2. Participants and Procedure

Participants were recruited over a period of six months from primary and secondary care ambulatory services in the “Hospital Universitario Fundación Jiménez Díaz” (HUFJD) healthcare network. This network comprises care ambulatory centers and a general hospital with a catchment area of 300,000 people.

Eligible participants were required to meet two criteria: (a) being older than 18 years old and (b) presenting themselves for a psychiatric consultation, either self-referred or referred by a GP. Consecutive sampling was employed, as we intended to screen mental health patients in the routine care workflow of psychiatric units.

The process of recruitment consisted in inviting the patients referred by the GP and attending consultation at the psychiatric units of the UH FJD during their first visit or in a subsequent visit (i.e., at the first instance that we could apply the E-mwTool). A clinician, a clinical psychologist, or a psychiatrist informed eligible participants of the study. After obtaining informed consent, participants completed the E-mwTool and other screening tools on their smartphones. The MINI-Plus was administered by the clinician thereafter on the same visit.

Participants were patients seen in consultation by a psychiatrist either on their first visit or in a subsequent visit (i.e., at the first instance that we could apply the E-mwTool). Clinical data were recorded during their routine healthcare visits.

Ethical approval was received from the “Instituto de Investigación Sanitaria de la Fundación Jiménez Díaz” (IIS-FJD: code ER1_PIC 76-2013_FJD). Informed consent was obtained from each participant before recruitment.

### 2.3. Materials and Measures

Between June and December 2019, psychiatrists received/phoned patient visits as part of their routine in which research psychologists conduct MINI-Plus interviews. The MINI-Plus is a structured diagnostic interview that has been widely adopted and can be simply administered in approximately 15 min [38,39,40]. It has been used as a reference standard across many contexts [41].

Participants were provided a mobile app with a built-in questionnaire, the E-mwTool, to screen patients’ mental health and classify them into treatment categories, as well as five self-administered questionnaires: the single-item drug use screening test [31]; the AUDIT-3 [32], the C-SSRS [33,34], the GAD-7 [35], and the PHQ-9 [36]. We also evaluated the performance of the original E-mwTool and the AUDIT-3 with two different response options and cutoffs.

The E-mwTool is a validated instrument that was developed by identifying a small number of items from a battery of mental health screeners via least absolute shrinkage and selection operator (LASSO) regression. The items that could best predict the presence of a wide range of mental disorders, as well as classify an individual into treatment-oriented disorder categories, were selected [29]. This brief, 2-step 12-item screener screens for severe mental disorders (including psychosis and mania), common mental disorders (including depression, anxiety, and post-traumatic disorder (PTSD)), alcohol use disorders, and suicide risk (see Table 1).

The original E-mwTool does not have an item dedicated to drug use, because the sample used to develop the tool had a very low prevalence of substance use disorders. Therefore, in this sample, we explored if a 13th item focused on illicit drug use, taken from a single-item drug use screening test with high sensitivity and specificity [31], would improve screening for substance use disorders when added to the E-mwTool.

### 2.4. Analytical Strategy

After anonymization, all analyses were performed by an independent investigator (C.B.). We first used descriptive statistics to describe baseline characteristics, sum scores of the screening instruments, and the prevalence of psychiatric disorders among study participants. To ascertain the predictive performance of the E-mwTool, we calculated sensitivity and specificity when compared to MINI-Plus diagnoses for any mental disorder and each of the four disorder categories (health dimensions in Table 1).

For the other screening instruments, we constructed nonparametric receiver operating characteristic curves (ROC curves) to examine the performance of each scale over a range of sum score cutoffs. We then calculated the area under the ROC curve (AUC), which is a global measure of the discriminating ability of a test [42]. It ranges from 0.5, representing no discrimination, to 1.0, representing perfect discrimination. Then, an optimal empirical cutoff point was identified via the Youden (J) index [42]. The J index combines the sensitivity and specificity of a cutoff in a single value, which is defined as follows: Sensitivity + Specificity—1. It ranges from −1 to 1, where the greater its value, the better the combined sensitivity and specificity of the cutoff point.

Point estimates for sensitivity, specificity, and AUC are presented along with their respective 95% confidence intervals. All analyses were performed on the set of participants with no missing MINI diagnoses or missing responses to the tool being evaluated. All statistical analyses were performed by using R version 4.0.5 [43].

## 3. Results

### 3.1. Population Characteristics and Prevalence of Mental Health Disorders

Table 2 presents the characteristics of the study population. A total of 433 participants had initially completed responses to all E-mwTool items and MINI diagnoses. Females (279, 64.4%) represented most of our study population, which had a mean age of 49.3 (standard deviation (SD) 16.3) years. According to the MINI-Plus, 313 (72.3%) participants had a mental health condition, with 58 participants (13.4%) having more than one diagnosis category. Common conditions were highly prevalent in our sample, whilst severe conditions or alcohol–drug disorders were relatively scarce (under 7%). Major depression—as both episodes and depressive disorder—was the most prevalent disorder. General anxiety disorder (107, 24.7%) was also prevalent in this sample.

### 3.2. Discriminating Capacity of E-mwTool

Table 3 shows the sensitivity and specificity of the 13-item E-mwTool for predicting MINI-Plus diagnoses. When looking at the first three items that serve as screening for any disorder, we observed excellent sensitivity of 0.97 (95% CI = 0.95–0.99). Sensitivity and specificity for detecting any common mental disorder were 0.96 (95% CI = 0.93–0.98) and 0.31 (95% CI = 0.24–0.39), respectively. Similarly, the E-mwTool showed high sensitivity for almost every common mental disorder, ranging from 0.90 (95% CI = 0.56–1.00) for social anxiety disorder to 1.0 (95% CI = 0.92–1.00) for panic disorder. As expected, specificity for these common disorders was low: from 0.13 (95% CI = 0.11–0.17) for social anxiety disorder to 0.19 (95% CI = 0.14–0.24) for major depressive episodes; specificities ranged from 0.13 to 0.19.

The E-mwTool showed excellent specificity (0.85, 95% CI = 0.82–0.89) for any severe mental disorder, but low sensitivity (0.21, 95% CI = 0.08–0.40). The E-mwTool performed well for suicide risk, with a sensitivity of 0.93 (95% CI = 0.76–0.99) and specificity of 0.60 (95% CI = 0.55–0.65); for alcohol use disorder, 0.86 (95% CI = 0.57–0.98); and substance use disorder, 0.73 (95% CI = 0.45–0.92). The complete description of the performance of the E-mwTool across different cutoffs for the AUDIT item is available in Supplementary File S1 online.

### 3.3. Discriminating Capacity and Optimal Cutoff Points of Self-Administered Questionnaires

Results from ROC analyses showed good discriminatory power for all the self-administered questionnaires. The PHQ-9 had an AUC of 0.78 (95% CI = 0.74–0.82) for major depression (depressive episodes or depressive disorder) diagnosis, but also had a strong performance for any common mental disorder (AUC = 0.75; 95% CI = 0.70–0.80) and any disorder (i.e., severe mental disorders, substance/alcohol use disorders, suicide risk; AUC = 0.71; 95% CI = 0.66–0.77). The optimal cutoff for the PHQ-9 predicting MINI depression according to Youden’s J was ≥10, with a sensitivity of 0.82 and a specificity of 0.58.

The GAD-7 had an AUC of 0.69 (95% CI = 0.65–0.74) for any anxiety-related disorder (social anxiety disorder, generalized anxiety disorder, panic, agoraphobia, OCD, or PTSD) and had similar results regarding common conditions (AUC = 0.73; 95% CI = 0.68–0.79) or any disorder (AUC = 0.69; 95% CI = 0.64–0.75). The optimal cutoff for the GAD-7 predicting MINI anxiety according to Youden’s J was ≥8, with a sensitivity of 0.87 and a specificity of 0.42.

The CSSR-S had also a good discriminating power for suicide risk (AUC = 0.79; 95% CI = 0.72–0.86), with an optimal cutoff of ≥”Low Risk” according to Youden’s J, with a sensitivity of 0.96 and a specificity of 0.59. The AUDIT-3 had a greater AUC (0.90; 95% CI = 0.82–0.97) regarding alcohol abuse, which decreased to 0.76 (95% CI = 0.65–0.86) when applied as a predictor of either alcohol or substance use disorder. For predicting alcohol use disorder, the optimal cutoff according to Youden’s J was ≥6, with a sensitivity of 0.71 and a specificity of 0.95. The single-question screening test for drug use in primary care performed well for substance use (AUC = 0.81, 95% CI = 0.72, 0.90). When considering possible cutoffs for this single-item measure, a cutoff of ≥1 maximized combined sensitivity and specificity (sensitivity = 0.67, specificity = 0.92).

A complete description of the sensitivity and specificity of each scale at every cutoff point, including the optimal cutoff points based on the Youden index, is available in Supplementary File S2.

## 4. Discussion

The primary aim of the present study was to examine the validity of the self-administered E-mwTool compared to the MINI-Plus diagnoses (criterion standard) in a general Spanish clinical population in HUFJD. Thus, it was hypothesized the E-mwTool could be a valid screening option based on its performance in screening a broad range of disorder categories.

In this sample collected during COVID-19, the majority of individuals were diagnosed with a common mental health condition. The usual diagnosis was major depression or general anxiety, which coincides with what other studies have reported during the pandemic [44]. The performance of the E-mwTool screening these disorders was strong, with a sensitivity of 0.96 for common mental disorders overall. Similarly, the sensitivity of the tool for suicide risk and alcohol/substance use disorders was high, with sensitivities of 0.93 and 0.79, respectively. The strong sensitivity to these conditions is especially relevant, as the need to detect suicide risk has increased along with the increase in suicide during and after the pandemic crisis [45,46].

However, for severe mental health conditions, in general, the sensitivity of the E-mwTool was very low for the disorders studied (e.g., bipolar disorders I and II, hypomania, and manic episodes, except those linked to psychotic disorders), though it is important to note that only a few patients were positively diagnosed with these other types of health problems.

While these findings support that the newly adapted E-mwTool has an excellent capacity to identify with high sensitivity most mental disorders in this clinical Spanish population, the E-mwTool did excel at generating a negative result for patients who do not present with mental health conditions (i.e., specificity or “true negative rate”). Generally, the higher the sensitivity, the lower the specificity, as these performance metrics are dependent [47]. In the screening context, sensitivity is a priority, and in this study, the E-mwTool excellently detected any psychiatric disorder, common mental disorders, alcohol use disorder, and suicide risk.

The E-mwTool was developed for similar purposes as other short screening tools, such as the Psychiatric Diagnostic Screening Questionnaire (PDSQ) [48,49]. These tools are like diagnostic aids designed to be used in clinical practice by psychiatric outpatients presenting for diagnosis and treatment and can facilitate the efficiency of conducting initial prediagnostic evaluations. According to Zimmerman and Mattia in 2001 [49,50], it is crucial that a diagnostic aid have good sensitivity to detect more cases, at least in the initial stage of the clinical process (i.e., screening, assessments, and intervention). Indeed, in comparison with the PDSQ, the E-mwTool has been validated and compared with other scales; more importantly, it has technologized the administration of the screening test through a mobile health application.

Concerning the previous E-mwTool studies, the findings are quite similar. For instance, in its first sample [2], when it was developed as a mHealth app and applied in Mozambique into primary care settings, there was an excellent sensitivity in the common mental disorders (94% vs. 96% in the present study). However, the subsequent ten items measuring the severe disorders plus substance and suicide risk had a fair to excellent specificity (63–93% vs. 60–85% in the present study). Similarly, the second study [2] tested the tool to create a future toolkit to help in screening and identifying the presence of these disorders in community samples during annual medical visits to support the healthcare system managed by this African country [29].

Thus, our results suggest that this new tool can be used as a screener to support populations experiencing the risk of a psychiatric disorder. Specifically, it would be useful as a screener to be used before visiting the outpatient clinic to support the prediagnosis of a mental health problem. There is already a need, highlighted in COVID-19 times, to support people with a high risk of psychological morbidity through enhancing the awareness and diagnosis of mental disorders through online and smartphone technologies, especially in primary care and emergency units, apart from psychiatric units [51].

A recent systematic review showed that the negative psychological impact of the pandemic was significant and that it can be associated with an increase in internet searches related to fear, anxiety, and depression [52]. Indeed, during the pandemic, the reduced availability of mental health services has been associated with an increase in mental health burden, potentially related to an escalating number of untreated people with mental health problems. During the COVID-19 pandemic, an increased number of users of mental health apps was also reported compared to prior to the pandemic. Male adults, who are less likely to access mental health services, were more likely to launch mental health apps compared to females [53]. Importantly, in spite of the usage increase, user engagement remained minimal. These findings support utilizing an easy-to-use and brief mHealth screening app, such as the E-mwTool, to efficiently and rapidly prescreen the potential need for mental health treatment either via self-report or during a visit to the GP. Engagement and adherence to mental health treatments would require referral to appropriate mental health services.

Initiatives such as the E-mwTool can mitigate how poorly prepared we were for the pandemic to continue the delivery of psychiatric services worldwide at a moment when the citizens needed more of this type of attention, and to help prevent the risks of severe disorders such as addictions and suicide [54]. Interestingly, while social distance measures were taken to avoid infections, it seems the reduction in human contact has shown a potential adverse outcome on suicide risk during the pandemic, and strategies to reduce the barriers to mental health treatment are paramount in these times [55].

The secondary goal of this study was to evaluate the five self-administered questionnaires in the Spanish community population. The evaluation of these screeners across possible sum score cutoffs allows clinicians to adapt their use to different scenarios, maximizing sensitivity or specificity or balancing the two; this is essential for making good clinical decisions on the basis of proposed screening tests, as in medical practice the decision threshold is crucial for usefulness [48]. Overall, the five self-administered questionnaires showed good discriminatory power for major depression, anxiety-related disorders, and the rest of the common and severe conditions, with AUC values mostly above 0.75. The findings from this study support the strong performance of these questionnaires in their short versions [33,34,35,36] and validate these tools in the Spanish language for psychiatric clinic outpatients during the COVID-19 pandemic.

While some of the optimal cutoffs for these screening tools determined by Youden’s J (combined sensitivity and specificity) are similar to those commonly used, other results suggest possible alterations to common practice. In past UHFJD psychiatric settings, a cutoff of ≥10 was used for the PHQ-9 and GAD-7 to screen for depression and anxiety, respectively. The results from this analysis suggest that the cutoff of ≥10 does maximize sensitivity and specificity for the PHQ-9, but a lower cutoff of ≥8 for the GAD-7 provides a higher sensitivity with less decrease in specificity. The CSSR-S cutoff of ≥”Low Risk” which is often used in UHFJD screening for suicide risk was supported by our data. However, the optimal AUDIT-C cutoff of ≥6 based on this analysis is slightly lower than the cutoff of ≥7 used in UHFJD screening practice. Lowering the commonly used cutoff to ≥6 may detect more individuals with alcohol use disorder, while minimally increasing false positives. Providing this information to clinicians allows them to make appropriate decisions regarding what cutoff to use given the needs of their patient population and their resources [48].

Regarding the limitations of this study, the sample could not be randomized due to the ethics procedure established by the IISFJD at UHFJD. As a result, there was no counterbalance between the order in which the E-mwTool and the MINI-Plus were administered (all individuals first answered the E-mwTool, and then clinicians administered the MINI-Plus); therefore, there was no opportunity to evaluate whether the order may have had an effect. However, the E-mwTool was intended as a screening measure, which is why it was administered before the MINI diagnosis process. Secondly, the representativeness of the sample should be taken cautiously as a snowball sampling approach was used, and future replications with larger samples and other sampling strategies can provide a major generalization of the findings. Thirdly, the psychiatric outpatient clinic is a private setting, which during pandemic times most commonly served individuals with mood and anxiety disorders and had few individuals with severe disorders. Finally, the mHealth app was administered to adults who were on average in late middle age, so it would have been optimal to perform a digital competency test to ensure their capacity to use the app. However, all were accepted and finally managed to use the app with no difficulties.

Our intended target population was suspected cases of mental health conditions and related disorders that may be seen in a GP (i.e., primary care physician) visit in an urban, high-income country context. The sample was recruited at secondary care centers when patients were already referred by GPs, so relevant variables that could have biased the proportion and distribution of mental health disorders in our sample could not be measured. For example, race; religion; socioeconomic status; institutional racism; availability, accessibility, and quality of primary healthcare; attrition in referral; and distressing COVID-19 circumstances have not been analyzed. Our study findings may not generalize to the broader population of suspected cases of mental health disorders seen by urban primary and secondary care teams in high-income countries.

## 5. Conclusions

In conclusion, this first examination of the E-mwTool with a Spanish sample during the pandemic indicates it may be a promising tool that can be beneficial under time constraints and COVID-19 measures (such as social distancing). Its screening will generally work well in detecting any psychiatric disorder, common psychiatric disorders, suicide risk, and alcohol/substance use disorders. However, as currently constructed, it has low sensitivity for severe disorders. Additionally, the tool has low overall specificity for most disorders, which should inform its use as a first-step screener rather than a tool for diagnosing patients. Overall, compared with the MINI-Plus, the new E-mwTool and the short versions of the self-questionnaires seem to detect those patients with psychiatric diseases quite well in a rapid and easy format at the front line of primary care. Given the psychological barriers to the diagnosis of mental disorders that exist among many adults attending their GPs, the E-mwTool might be a good first step in facilitating subsequent help-seeking among the general population reluctant to engage in other types of screening.

## Figures and Tables

**Table 1:** Health dimensions and the number of questions of E-mwTool and self-administered questionnaires.

Questionnaire	Health Dimension	Number of Questions
Electronic Mental Wellness Tool (E-mwTool)	Common mental disorder	3
	Severe mental disorder	4
	Suicide risk	3
	Alcohol use disorder	2
	Illegal drug use or nonprescribed medication	1
Alcohol Use Disorders Identification Test, short version (AUDIT-3)	Alcohol use	3
Columbia-Suicide Severity Rating Scale (C-SSRS)	Suicide behavior	7
Generalized Anxiety Disorder-7 (GAD-7)	Anxiety	7
Patient Health Questionnaire-9 (PHQ–9)	Depression	9

**Table 2:** Descriptive characteristics of participants (n = 433).

Variable	n (%) or Mean (SD)
**Age**	49.3 (16.3)
**Gender**	
Female	279 (64.4%)
Male	153 (35.3%)
Transgender	1 (0.2%)
**Country**	
Spain	358 (86.3%)
Other	57 (13.7%)
**Concurrent conditions**	
Patients with No Diagnosis	120 (27.7%)
Patients with 1 Diagnosis Category	255 (58.9%)
Patients with >1 Diagnosis Category	58 (13.4%)
**Diagnosis Categories**	
MINI-Plus Common	288 (66.5%)
Major Depressive Episode	151 (34.9%)
Major Depressive Disorder	91 (21.0%)
Panic Disorder	45 (10.4%)
Agoraphobia	25 (5.8%)
Social Anxiety Disorder	10 (2.3%)
OCD	16 (3.7%)
PTSD	35 (8.1%)
Generalized Anxiety Disorder	107 (24.7%)
MINI-Plus Severe	29 (6.7%)
Manic Episode	0 (0%)
Hypomania	2 (0.5%)
Bipolar Type I	7 (1.6%)
Bipolar Type I w/Psychotic Symptoms	1 (0.2%)
Bipolar Type II	3 (0.7%)
Psychotic Disorder	17 (3.9%)
MDD w/Psychotic Symptoms	1 (0.2%)
Suicide risk	27 (6.2%)
Alcohol/Substance	29 (6.7%)
Alcohol use disorder	14 (3.2%)
Substance use disorder	15 (3.5%)

Abbreviations: OCD = obsessive–compulsive disorder; PTSD = post-traumatic stress disorder; MDD = manic–depressive disorder.

**Table 3:** Performance of 13-item E-mwTool in identification and classification of participants with mental disorders (n = 433).

Disorder	N	%	Sensitivity	95% CI	Specificity	95% CI
**Common mental disorders**	288	67	0.96	0.93–0.98	0.31	0.24–0.39
Agoraphobia	25	5.8	1.00	0.86–1.00	0.14	0.11–0.18
Generalized Anxiety Disorder	107	24.7	0.97	0.92–0.99	0.17	0.13–0.21
Major Depressive Disorder	91	21	0.98	0.92–1.00	0.16	0.12–0.20
Major Depressive Episode	151	34.9	0.97	0.93–0.99	0.19	0.14–0.24
Obsessive–Compulsive Disorder	16	3.7	1.00	0.79–1.00	0.14	0.10–0.17
Panic Disorder	45	10.4	1.00	0.92–1.00	0.15	0.11–0.19
Post-traumatic stress disorder	35	8.1	0.91	0.77–0.98	0.14	0.10–0.17
Social Anxiety Disorder	10	2.3	0.90	0.56–1.00	0.13	0.10–0.17
**Severe mental disorders**	29	6.7	0.21	0.08–0.40	0.85	0.82–0.89
Bipolar Type I	7	1.6	0.14	0.00–0.58	0.85	0.81–0.88
Bipolar Type I w/Psychotic Symptoms	1	0.2	1.00	0.03–1.00	0.85	0.82–0.88
Bipolar Type II	3	0.7	0.33	0.01–0.91	0.85	0.81–0.88
Hypomania	2	0.5	0.00	0.00–0.84	0.85	0.81–0.88
Manic Episode	0	0.0	-	-	-	-
MDD w/Psychotic Symptoms	1	0.2	1.00	0.03–1.00	0.85	0.82–0.88
Psychotic Disorder	17	3.9	0.18	0.04–0.43	0.85	0.81–0.88
**Substance/Alcohol Use Disorders**	29	6.2	0.79	0.60–0.92	0.77	0.73–0.81
Alcohol	14	6.2	0.86	0.57–0.98	0.77	0.73–0.81
Substance	15	3.2	0.73	0.45–0.92	0.67	0.62–0.71
**Suicide Risk**	27	6.2	0.93	0.76–0.99	0.60	0.55–0.65

Abbreviations: OCD = obsessive–compulsive Disorder; PTSD = post-traumatic stress disorder, MDD = manic–depressive disorder.

## Data Availability

Data sharing is not applicable to this article.

## Supplementary Materials

The following supporting information can be downloaded at: https://www.mdpi.com/article/10.3390/ijerph20043204/s1, Supplementary File S1: Performance of E-mwTool for substance/alcohol use at varied AUDIT item 1 cutoffs; Supplementary File S2: Optimal cutoff points and summary of performance of self-administered questionnaires across all cutoff points.
