# Supplementary material for: The Electronic Mental Wellness Tool as a Self-Administered Brief Screening Instrument for Mental Disorders in the General Spanish Population during the Post-COVID-19 Era
Source: Int J Environ Res Public Health. 2023 Feb 11;20(4):3204. doi: 10.3390/ijerph20043204 (PMC9959534; doi:10.3390/ijerph20043204)
Supplement: Supplementary file 1 [file ijerph-20-03204-s001.zip › ijerph-2148924-supplementary.pdf]

Supplementary File S1

Performance of E-mwTool for substance/alcohol use at varied AUDIT item 1 cutoffs.

MwTool with additional substance use item

Individual Disorder performance (n = 433)

	Proportion Positive (n)	Sensitivity	Specificity
Alcohol/Substance[a]	0.067 (29)	0.793 (0.603, 0.92)	0.772 (0.728, 0.812)
Alcohol use disorder	0.032 (14)	0.857 (0.572, 0.982)	0.754 (0.710, 0.795)
Substance use disorder	0.035 (15)	0.733 (0.449, 0.922)	0.751 (0.707, 0.792)
Alcohol/Substance[b]	0.067 (29)	0.828 (0.642, 0.942)	0.668 (0.62, 0.714)
Alcohol use disorder	0.032 (14)	0.929 (0.661, 0.998)	0.654 (0.606, 0.699)
Substance use disorder	0.035 (15)	0.733 (0.449, 0.922)	0.648 (0.600, 0.694)

a. AUDIT 1 cutoff: >= "Once a week"
b. AUDIT 1 cutoff: >= "Once a month"

Supplementary File S2

Optimal cutoff points and summary of performance of self-administered questionnaires across all cutoff points.

Table S1. Summary of performance for optimal cut-off points based on Youden's J

Outcome	Cut-point	Se	Sp	PPV	NPV	LR+	LR-
AUDIT-3							
Alcohol/Substance Use Disorder	3.5	0.62	0.84	0.22	0.97	3.88	0.45
Alcohol Use Disorder	5.5	0.71	0.95	0.32	0.99	14.20	0.31
CSSR-S							
Suicide risk	1 (Low)	0.96	0.59	0.08	1.00	2.34	0.07
GAD-7							
Any MINI Disorder	12.5	0.5	0.77	0.85	0.37	2.17	0.65
Common MINI Disorder	7.5	0.77	0.57	0.78	0.55	1.79	0.40
Anxiety-related conditions	9.5	0.75	0.56	0.68	0.64	1.70	0.45
Anxiety-related conditions (without OCD)	10.5	0.72	0.62	0.67	0.67	1.89	0.45

Anxiety-related conditions (without PTSD)	7.5	0.86	0.46	0.59	0.78	1.59	0.30
Anxiety (SAD + GAD)	7.5	0.87	0.42	0.36	0.89	1.50	0.31
PHQ-9							
Any MINI Disorder	8.5	0.76	0.54	0.82	0.46	1.65	0.44
Common MINI Disorder	9.5	0.75	0.62	0.80	0.55	1.97	0.40
Major Depression (Disorder or Episode)	9.5	0.82	0.58	0.72	0.71	1.95	0.31

Se, Sensitivity; Sp, Specificity; PPV, Predictive Positive Value; NPV, Negative Predictive Value; LR+, Positive Likelihood Ratio; LR-, Negative Likelihood Ratio.

AUDIT-3: ROC Curve and cut-off points (n=428)

MINI Alcohol/Substance

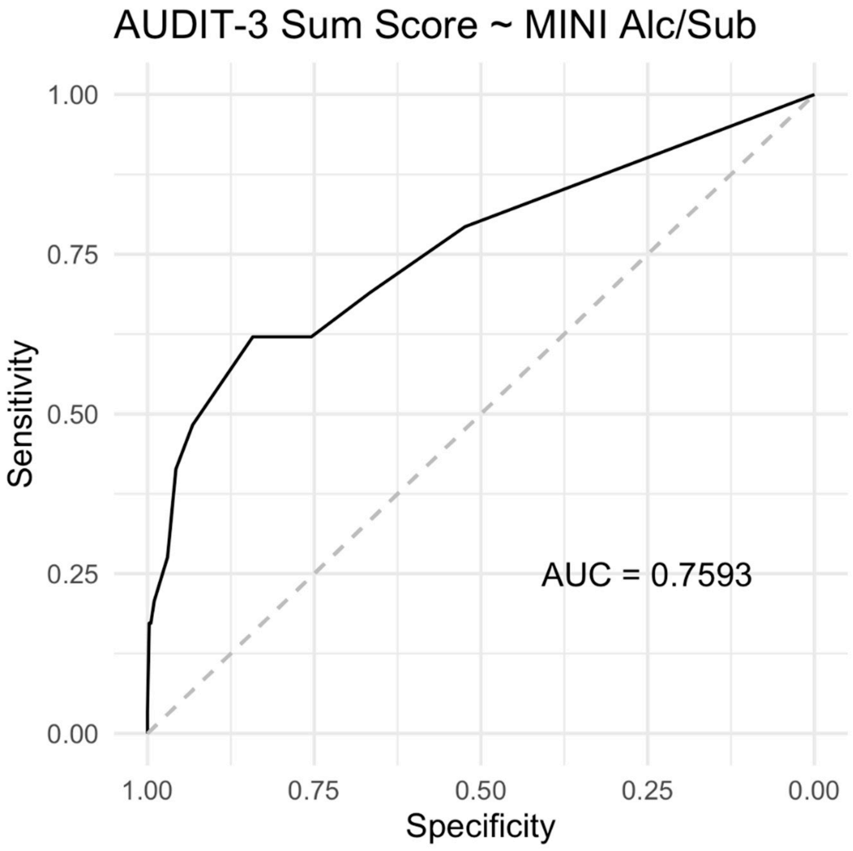

Threshold	Sensitivity	Specificity
0.5	0.793	0.524
1.5	0.69	0.667
2.5	0.621	0.754
3.5	0.621	0.842
4.5	0.483	0.932
5.5	0.414	0.957
6.5	0.276	0.97
7.5	0.207	0.99
8.5	0.172	0.995
9.5	0.172	0.997
10.5	0.034	1

Highlight indicates cutoff with maximum Youden's index

Youden's Index = (Sensitivity + Specificity - 1)

MINI Alcohol

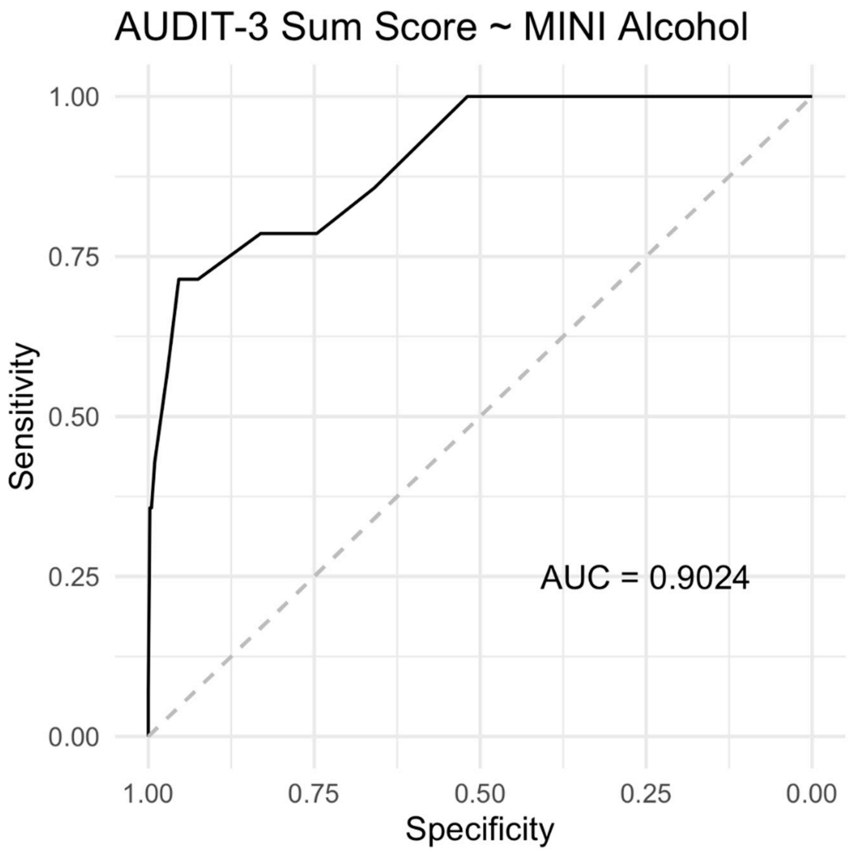

Threshold	Sensitivity	Specificity
0.5	1	0.519
1.5	0.857	0.659
2.5	0.786	0.746
3.5	0.786	0.831
4.5	0.714	0.925
5.5	0.714	0.954
6.5	0.571	0.971
7.5	0.429	0.99
8.5	0.357	0.995
9.5	0.357	0.998
10.5	0.071	1

Highlight indicates cutoff with maximum Youden's index

C-SSRS: ROC Curve and cut-off points (n=427)

MINI Suicide

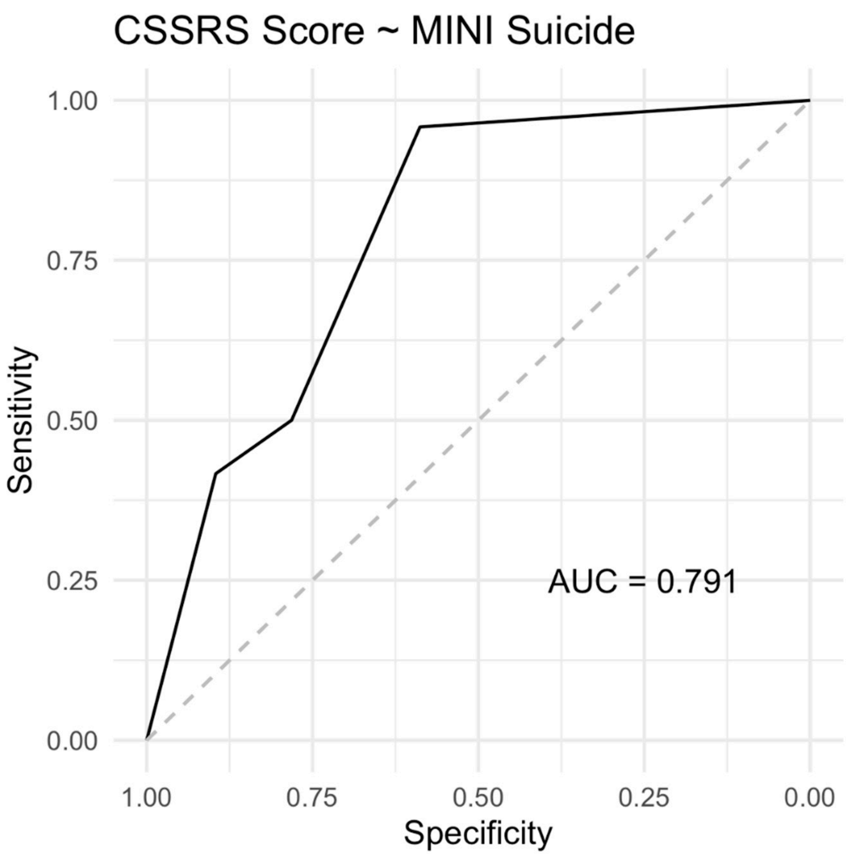

Threshold	Sensitivity	Specificity
>= Low	0.958	0.588
>= Medium	0.5	0.782
>= High	0.417	0.896

Highlight indicates cutoff with maximum Youden's index
Youden's Index = (Sensitivity + Specificity - 1)

GAD-7: ROC Curve and cut-off points (n=428)

Any MINI Disorder

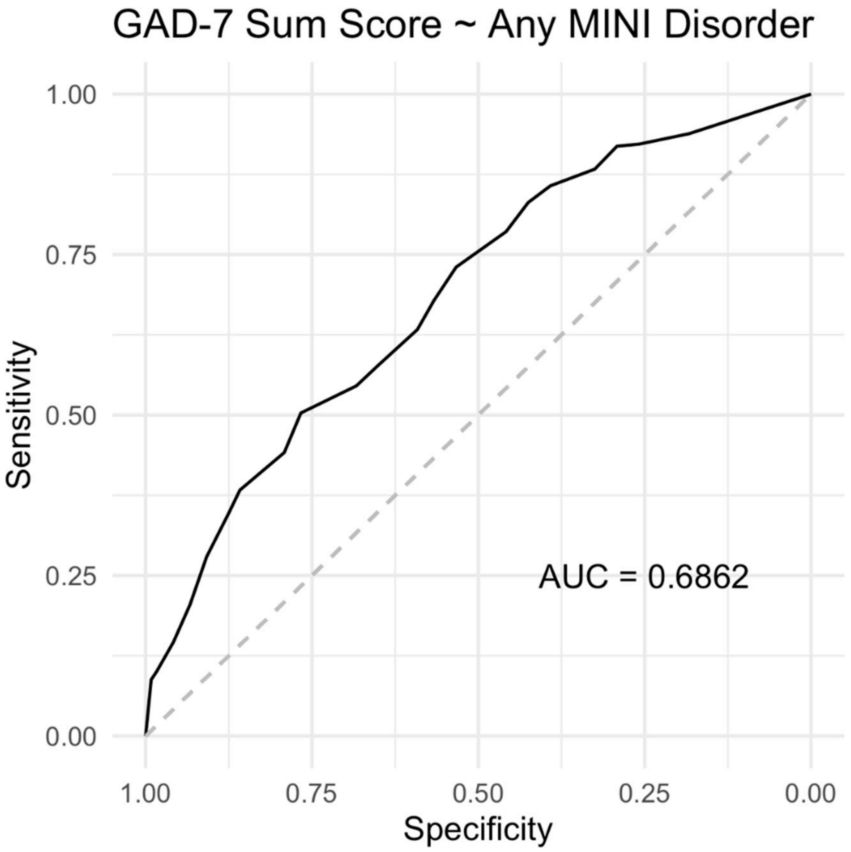

Threshold	Sensitivity	Specificity
0.5	0.938	0.183
1.5	0.922	0.258
2.5	0.919	0.292
3.5	0.883	0.325
4.5	0.857	0.392
5.5	0.831	0.425
6.5	0.786	0.458
7.5	0.731	0.533
8.5	0.679	0.567
9.5	0.633	0.592
10.5	0.578	0.65
11.5	0.545	0.683
12.5	0.503	0.767
13.5	0.442	0.792
14.5	0.383	0.858
15.5	0.347	0.875

16.5	0.279	0.908
17.5	0.205	0.933
18.5	0.146	0.958
19.5	0.101	0.983
20.5	0.088	0.992

Highlight indicates cutoff with maximum Youden's index
Youden's Index = (Sensitivity + Specificity - 1)

MINI Common Disorder

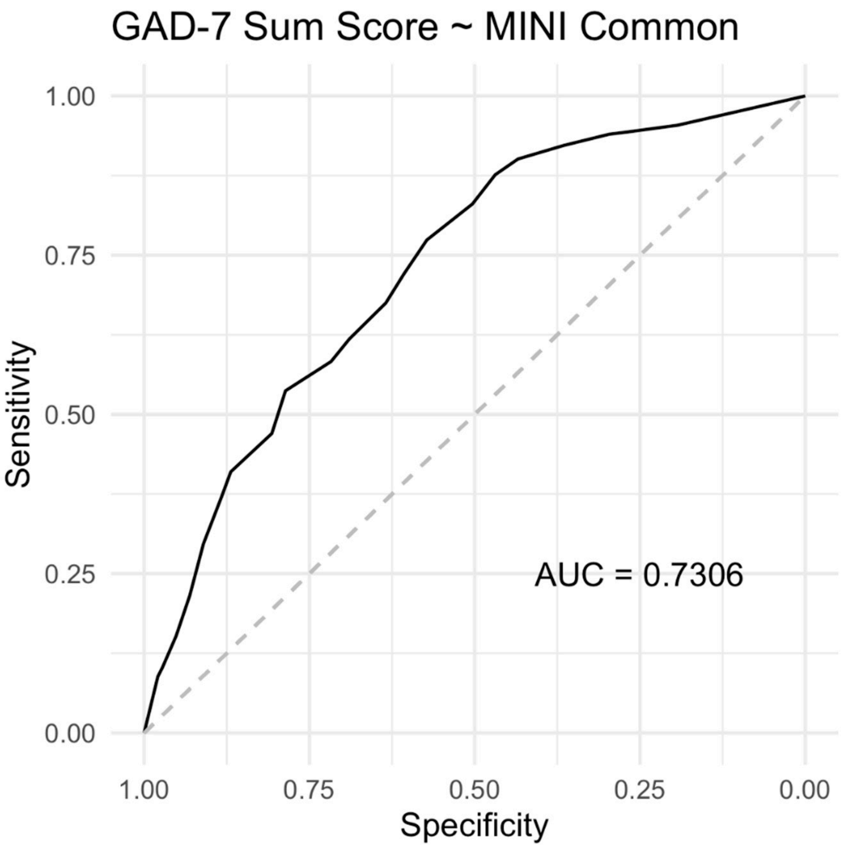

Threshold	Sensitivity	Specificity
0.5	0.954	0.193
1.5	0.943	0.269
2.5	0.94	0.297
3.5	0.922	0.366
4.5	0.901	0.434
5.5	0.876	0.469
6.5	0.83	0.503
7.5	0.774	0.572
8.5	0.721	0.607
9.5	0.675	0.634
10.5	0.618	0.69
11.5	0.583	0.717
12.5	0.537	0.786
13.5	0.47	0.807
14.5	0.41	0.869
15.5	0.371	0.883

16.5	0.297	0.91
17.5	0.216	0.931
18.5	0.152	0.952
19.5	0.102	0.972
20.5	0.088	0.979

Highlight indicates cutoff with maximum Youden's index

MINI Anxiety (All)

Social Anxiety Disorder, Generalized Anxiety Disorder

Panic, Agoraphobia, OCD, PTSD

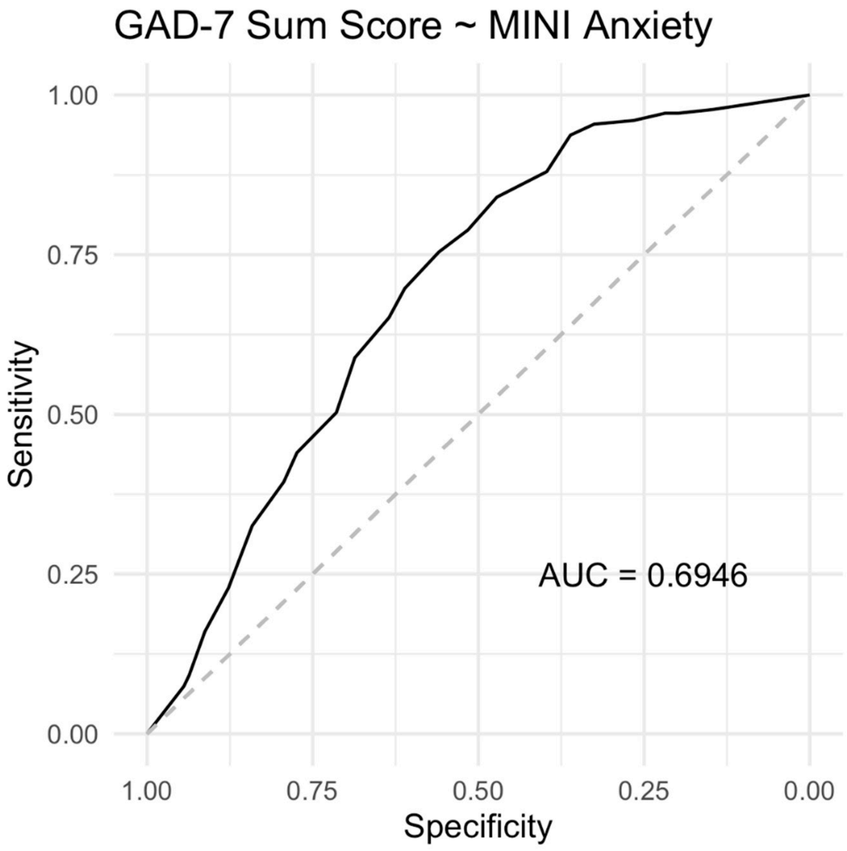

Threshold	Sensitivity	Specificity
0.5	0.977	0.147
1.5	0.971	0.198
2.5	0.971	0.218
3.5	0.96	0.266
4.5	0.954	0.325
5.5	0.937	0.361
6.5	0.88	0.397
7.5	0.84	0.472
8.5	0.789	0.516
9.5	0.754	0.56
10.5	0.697	0.611
11.5	0.651	0.635
12.5	0.589	0.687

13.5	0.503	0.714
14.5	0.44	0.774
15.5	0.394	0.794
16.5	0.326	0.841
17.5	0.229	0.877
18.5	0.16	0.913
19.5	0.091	0.937
20.5	0.074	0.944

Highlight indicates cutoff with maximum Youden's index

MINI Anxiety (no OCD)

Social Anxiety Disorder, Generalized Anxiety Disorder

Panic, Agoraphobia, PTSD

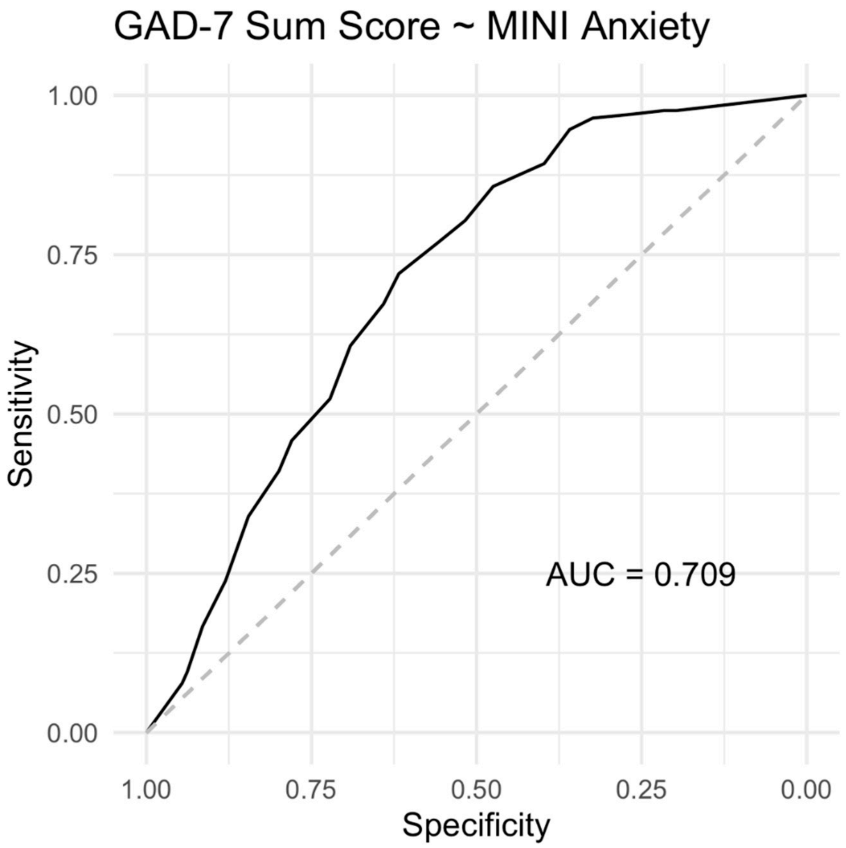

Threshold	Sensitivity	Specificity
0.5	0.982	0.147
1.5	0.976	0.197
2.5	0.976	0.216
3.5	0.97	0.266
4.5	0.964	0.324
5.5	0.946	0.359
6.5	0.893	0.398
7.5	0.857	0.475
8.5	0.804	0.517
9.5	0.768	0.56
10.5	0.72	0.618
11.5	0.673	0.641
12.5	0.607	0.691
13.5	0.524	0.722
14.5	0.458	0.78
15.5	0.411	0.799

16.5	0.339	0.846
17.5	0.238	0.88
18.5	0.167	0.915
19.5	0.095	0.938
20.5	0.077	0.946

Highlight indicates cutoff with maximum Youden's index

MINI Anxiety (no PTSD)

Social Anxiety Disorder, Generalized Anxiety Disorder

Panic, Agoraphobia, OCD

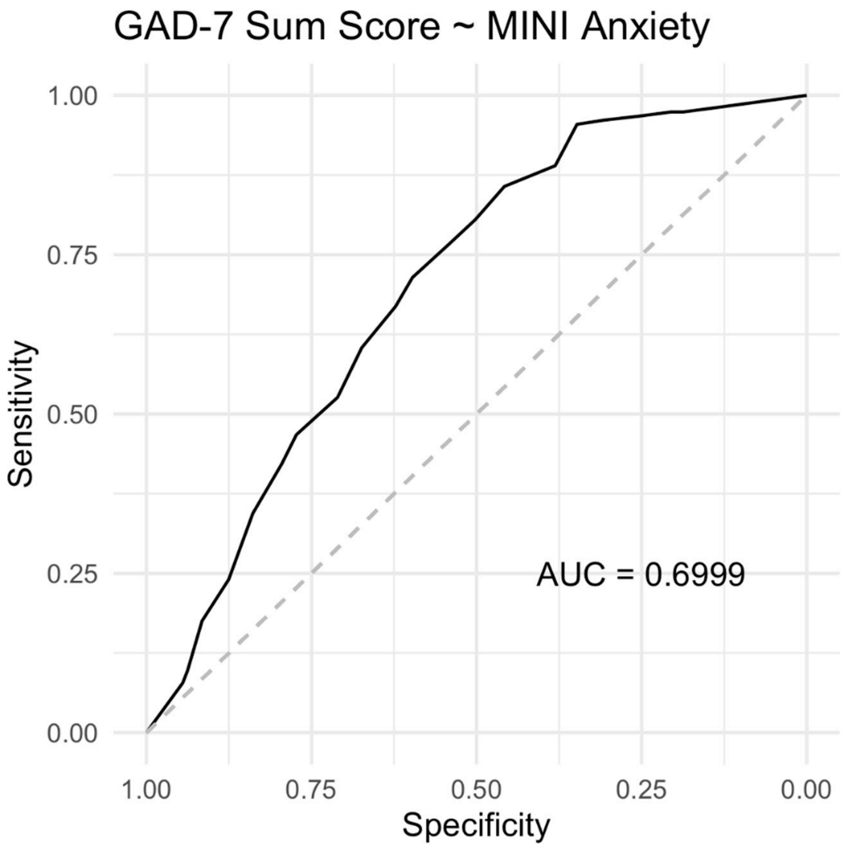

Threshold	Sensitivity	Specificity
0.5	0.981	0.139
1.5	0.974	0.187
2.5	0.974	0.205
3.5	0.968	0.253
4.5	0.961	0.308
5.5	0.955	0.348
6.5	0.89	0.381
7.5	0.857	0.458
8.5	0.805	0.502
9.5	0.766	0.542
10.5	0.714	0.597
11.5	0.669	0.623
12.5	0.604	0.674
13.5	0.526	0.711
14.5	0.468	0.773
15.5	0.422	0.795

16.5	0.344	0.839
17.5	0.24	0.875
18.5	0.175	0.916
19.5	0.097	0.938
20.5	0.078	0.945

Highlight indicates cutoff with maximum Youden's index

MINI Anxiety (SAD + GAD)
Social Anxiety Disorder or Generalized Anxiety Disorder

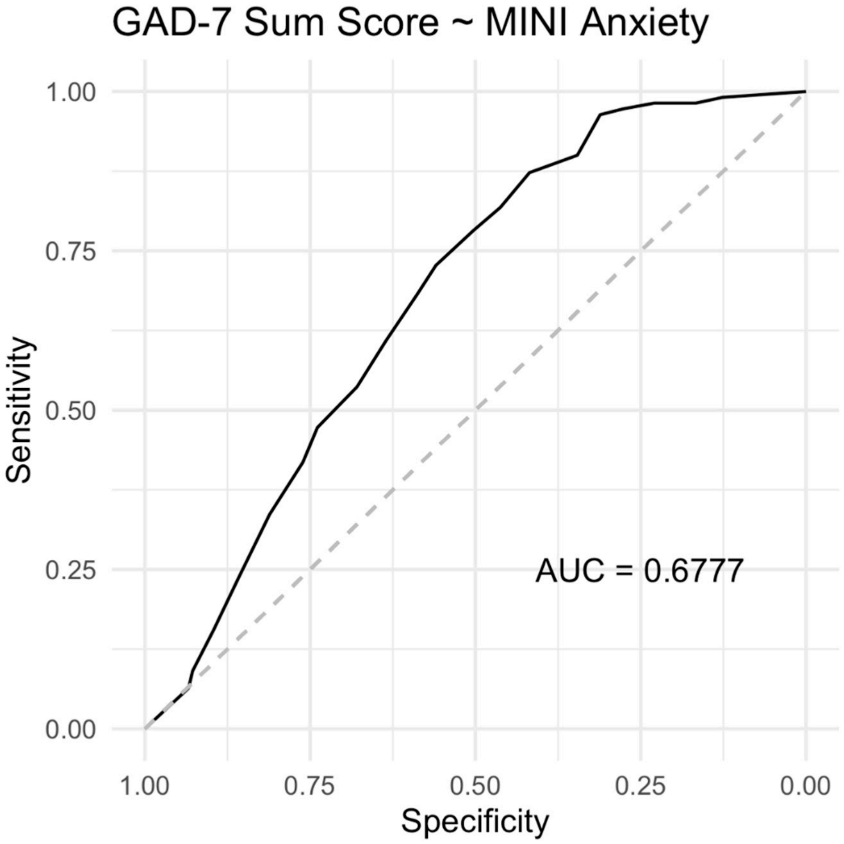

Threshold	Sensitivity	Specificity
0.5	0.991	0.126
1.5	0.982	0.167
2.5	0.982	0.182
3.5	0.982	0.23
4.5	0.973	0.277
5.5	0.964	0.311
6.5	0.9	0.346
7.5	0.873	0.418
8.5	0.818	0.462
9.5	0.782	0.503
10.5	0.727	0.56
11.5	0.682	0.588
12.5	0.609	0.635
13.5	0.536	0.679
14.5	0.473	0.739

15.5	0.418	0.761
16.5	0.336	0.811
17.5	0.236	0.858
18.5	0.155	0.896
19.5	0.091	0.928
20.5	0.064	0.934

Highlight indicates cutoff with maximum Youden's index

PHQ-9: ROC Curve and cut-off points (n=428)

Any MINI Disorder

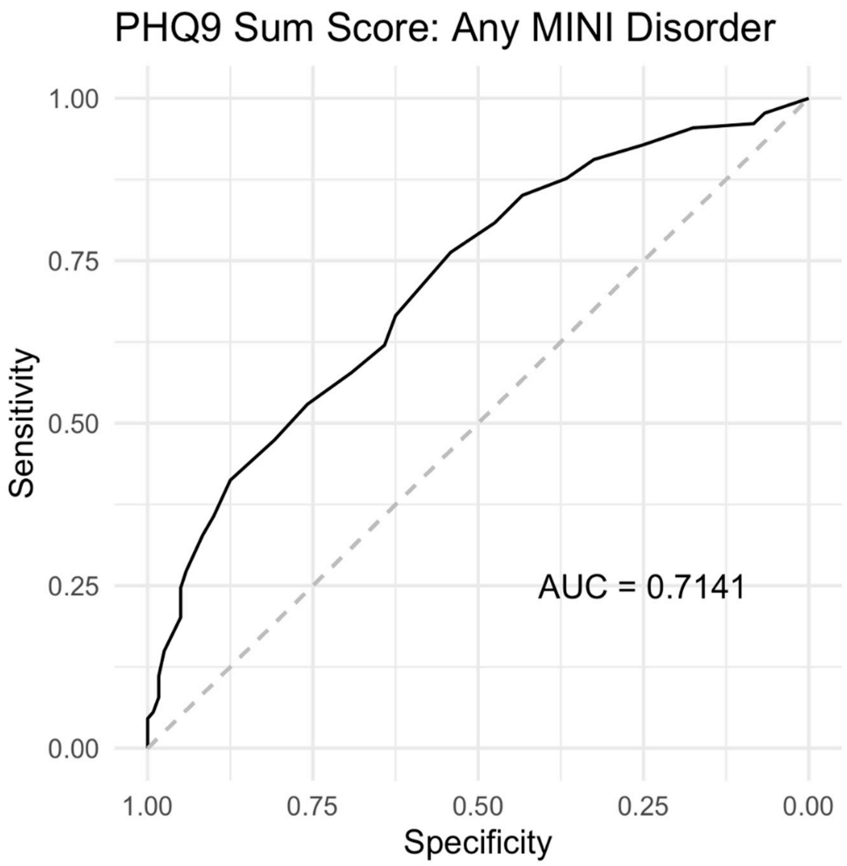

Threshold	Sensitivity	Specificity
0.5	0.977	0.067
1.5	0.961	0.083
2.5	0.955	0.175
3.5	0.929	0.25
4.5	0.906	0.325
5.5	0.877	0.367
6.5	0.851	0.433
7.5	0.808	0.475
8.5	0.763	0.542
9.5	0.705	0.592
10.5	0.666	0.625
11.5	0.62	0.642
12.5	0.578	0.692
13.5	0.529	0.758
14.5	0.474	0.808
15.5	0.412	0.875

	16.5	0.357	0.9
	17.5	0.328	0.917
	18.5	0.273	0.942
	19.5	0.247	0.95
	20.5	0.201	0.95
	21.5	0.149	0.975
	22.5	0.11	0.983
	23.5	0.078	0.983
	24.5	0.055	0.992
	25.5	0.045	1
	26.5	0.029	1

Highlight indicates cutoff with maximum Youden's index

Youden's Index = (Sensitivity + Specificity - 1)

MINI Common Disorder

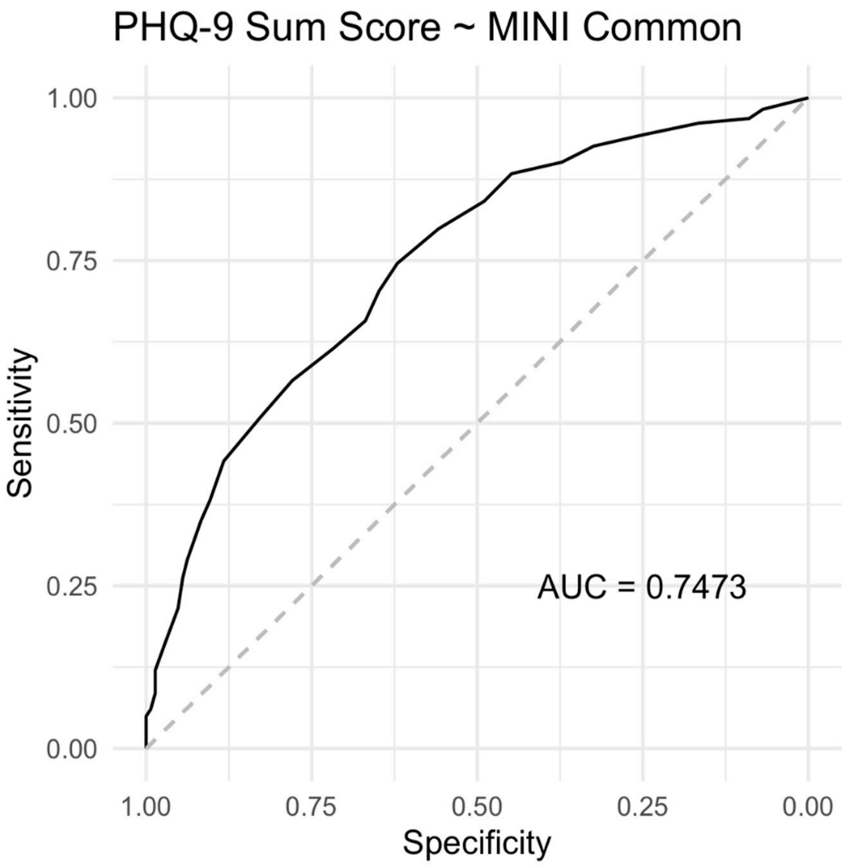

Threshold	Sensitivity	Specificity
0.5	0.982	0.069
1.5	0.968	0.09
2.5	0.961	0.166
3.5	0.943	0.248
4.5	0.926	0.324
5.5	0.901	0.372
6.5	0.883	0.448
7.5	0.841	0.49
8.5	0.799	0.559
9.5	0.746	0.621
10.5	0.703	0.648
11.5	0.657	0.669
12.5	0.615	0.717
13.5	0.565	0.779
14.5	0.509	0.828
15.5	0.442	0.883
16.5	0.382	0.903

17.5	0.35	0.917
18.5	0.29	0.938
19.5	0.261	0.945
20.5	0.216	0.952
21.5	0.159	0.972
22.5	0.12	0.986
23.5	0.085	0.986
24.5	0.06	0.993
25.5	0.049	1
26.5	0.032	1

Highlight indicates cutoff with maximum Youden's index

MINI Depression

Major Depressive Episode or Major Depressive Disorder

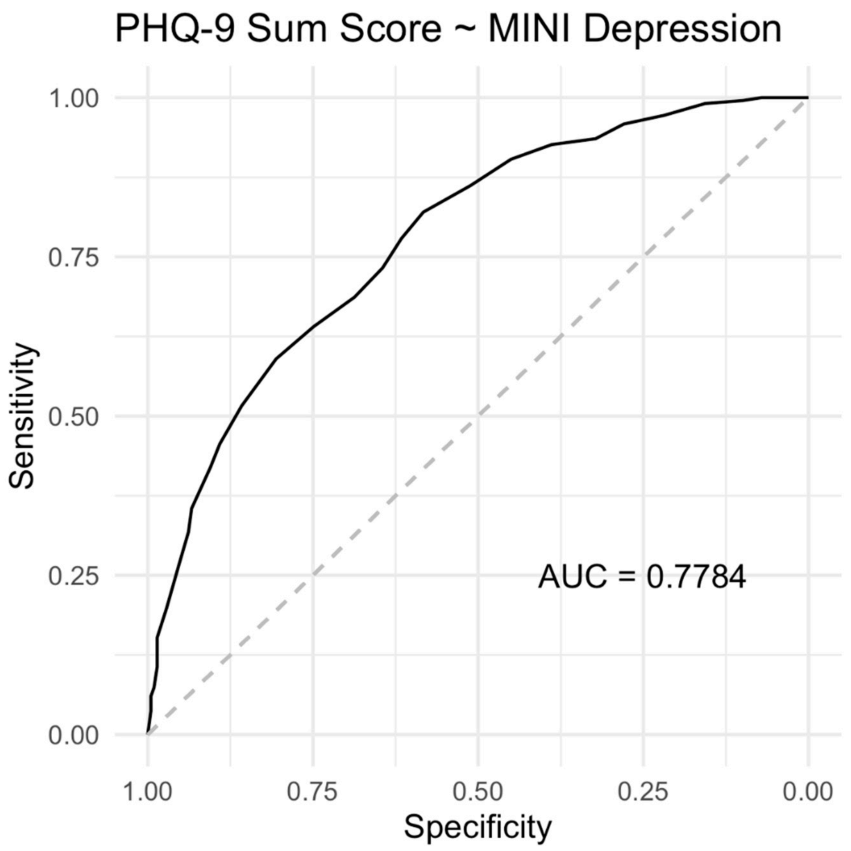

Threshold	Sensitivity	Specificity
0.5	1	0.071
1.5	0.995	0.1
2.5	0.991	0.156
3.5	0.972	0.218
4.5	0.959	0.28
5.5	0.935	0.322
6.5	0.926	0.389
7.5	0.903	0.45
8.5	0.862	0.512
9.5	0.82	0.583
10.5	0.779	0.616
11.5	0.733	0.645
12.5	0.687	0.687
13.5	0.641	0.749
14.5	0.59	0.806

15.5	0.516	0.858
16.5	0.456	0.891
17.5	0.419	0.905
18.5	0.355	0.934
19.5	0.318	0.938
20.5	0.267	0.953
21.5	0.198	0.972
22.5	0.152	0.986
23.5	0.106	0.986
24.5	0.074	0.991
25.5	0.06	0.995
26.5	0.037	0.995

Highlight indicates cutoff with maximum Youden's index

Single-item Substance Use Screener: ROC Curve and cut-off points (n=433)

Substance Use Disorder

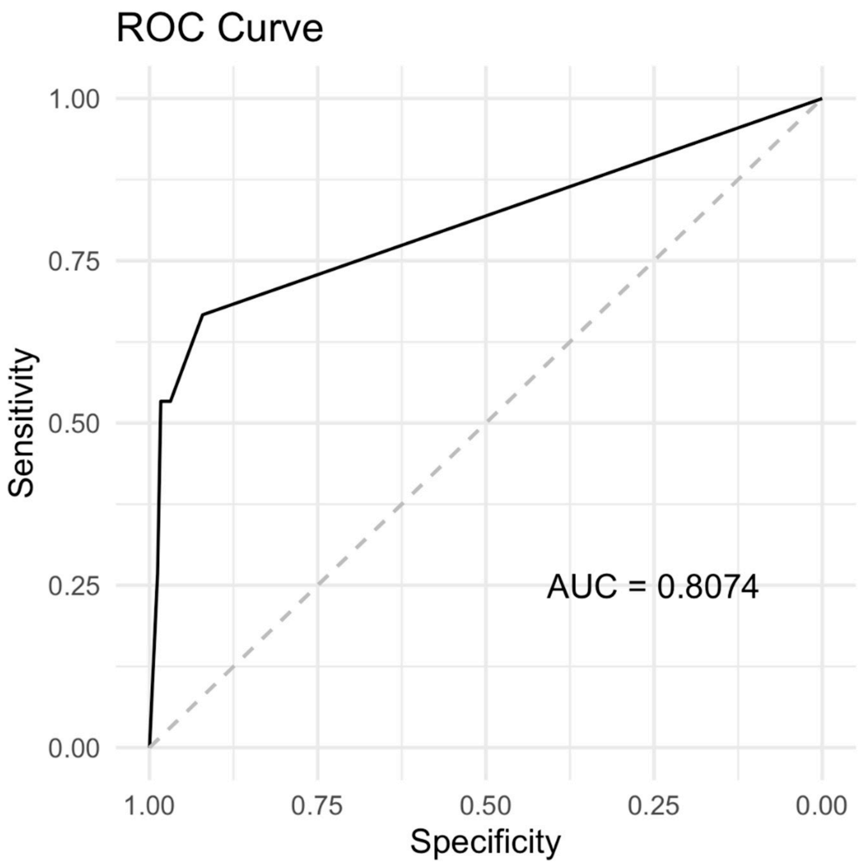

Cutoff	Sensitivity	Specificity
>= Once or twice (1)	0.667	0.921
>= Once a month (2)	0.533	0.969
>= Once a week (3)	0.533	0.983
>= Almost daily (4)	0.267	0.988

Highlight indicates cutoff with maximum Youden's index
